# Interaction effects of alcohol consumption and dizziness/vertigo on fall risk in psychiatric Inpatients: a cross-sectional study

**DOI:** 10.3389/fpsyt.2025.1653281

**Published:** 2026-01-08

**Authors:** Jing Peng, Huixia Liao, Qin Liu, Yuan Qin, Xubin He, Yanlin Gong, Tiaoxia Dong, Chengcheng Zhang, Jianghong Yu, Shengjun Wang, Juntao Zuo, Jianmei Long, Qingfang Yao, Bo Yang

**Affiliations:** 1 Department of Geriatrics Medicine, Jinzi Mountain Hospital of Chongqing Mental Health Center, Chongqing, China; 2 Department of Psychosomatic Sleep Medicine, Jinzi Mountain Hospital of Chongqing Mental Health Center, Chongqing, China; 3 Department of Psychiatry, GeLe Mountain Hospital of Chongqing Mental Health Center, Chongqing, China; 4 Department of Psychology, GeLe Mountain Hospital of Chongqing Mental Health Center, Chongqing, China; 5 Department of Nursing, Jinzi Mountain Hospital of Chongqing Mental Health Center, Chongqing, China

**Keywords:** alcohol, dizziness/vertigo, falls, interaction effect, psychiatric inpatients

## Abstract

**Background:**

Patients with mental disorders have a high incidence of falls and fall-related injuries, and although a history of alcohol consumption and dizziness/vertigo symptoms are assessed at the admission stage, their combined effect on fall risk has not been adequately quantified.

**Objectives:**

This exploratory study aimed to investigate the potential interaction between a history of alcohol consumption and dizziness/vertigo on the occurrence of falls among patients with mental disorders.

**Methods:**

A cross-sectional study was conducted among patients hospitalized in a psychiatric specialty hospital. Patients were divided into fall and non-fall groups. Data were analyzed using a Chi-square test and Multivariate logistic regression. Multiplicative and additive models were applied to calculate the interactions of a history of alcohol consumption and dizziness/vertigo, respectively.

**Results:**

2210 participants were included, 194 in the falls group and 2016 in the non-falls group. Multivariate analysis demonstrated that the utilization of antiepileptic medication, a history of alcohol consumption, dizziness/vertigo, body mass index (BMI) and diagnostic type were independent risk factors for falls in patients diagnosed with mental disorders. The multiplicative interaction analysis revealed a statistically significant interaction between a history of alcohol consumption and the presence of dizziness/vertigo on the incidence of falls among patients with mental disorders, after controlling for relevant variables(*P*<0.05). The additive interaction results suggested that the relative excess risk due to interaction (RERI), the attributable proportion due to interaction (API), and the synergy index (SI) were 7.470 (95% *CI*: 2.701-12.239), 0.674 (95% *CI*: 0.511-0.838), and 3.864 (95% *CI*: 2.040-7.320) after adjusting for relevant variables.

**Conclusion:**

There is a potential positive interaction between a history of alcohol consumption and dizziness/vertigo on the occurrence of falls in patients with mental disorders. Given the exploratory nature of this study and the small sample size of the co-exposed group, this conclusion requires validation in larger, prospective studies.

## 1 Introduction

Falls are the second leading cause of unintentional injury-related mortality globally (1). The consequences of falls encompass fractures, craniocerebral injuries, disability (2), secondary psychological trauma, such as helplessness and fear (3), and increased healthcare expenses (4). Individuals with mental disorders are particularly susceptible to falls. For example, certain studies have documented fall incidences ranging from 13 to 25 per 1,000 inpatient days—four to eight times higher than those observed in the general population (5, 6). This vulnerability that may be intensified by various factors, including sedative or orthostatic hypotensive effects of medications (e.g., antipsychotics, benzodiazepines), challenges in maintaining balance associated with psychomotor slowing, and behavioral changes such as impulsivity or impaired judgment (7). More critically, the risk of hip fracture following a fall in such patients is four times higher than in the general population, and the risk is further exacerbated when combined with cognitive impairment or delirium (8). Consequently, falls in patients with mental disorders represent a major public health issue.

Current research has focused intensely on independent predictors of fall risk, such as sedation and orthostatic hypotension induced by antipsychotic medications, as well as psychotic symptoms, all of which are associated with fall risk (9). However, falls typically result from a multifactorial interplay (10). Two key and often coexisting factors in individuals with mental disorders are a history of alcohol consumption and symptoms of dizziness/vertigo. alcohol consumption is markedly elevated among individuals with mental disorders (11). The prevalence of alcohol use disorders—encompassing both alcohol abuse and alcohol dependence—can reach up to 36.2 percent among those with severe mental disorders (12). Dizziness/vertigo—a core symptom of vestibular dysfunction that can be objectively quantified by measures such as the caloric test (13)—is frequently comorbid with mental disorders (14). This is because neural circuits associated with the vestibular nervous system are linked to emotional disorders such as anxiety and depression (15). This association parallels the bidirectional connection between tinnitus disorders and depression/anxiety (16): pre-existing emotional disorders intensify patients’ perception of dizziness/vertigo symptoms, while the dizziness/vertigo itself further exacerbates psychological distress, creating a vicious cycle. These two factors significantly increase the risk of falls by affecting balance and postural stability (17–19). However, traditional risk prediction models typically treat these factors as isolated variables, potentially obscuring synergistic effects and resulting in the failure to identify high-risk groups.

From a public health management perspective, identifying this biological synergy—statistically termed ‘interaction’—is crucial. Interaction refers to the phenomenon where the effect of one factor varies depending on the level of another factor, and the combined effect of two factors is not equivalent to the sum (additive interactions, INTA) or product (multiplicative interactions, INTM) of their individual effects (20). Rothman (21) proposed three metrics to assess interaction: the relative excess risk due to interaction (RERI), the attributable proportion due to interaction (API), and the synergy index (SI) to evaluate whether there is an additive interaction between factors. Additive interactions are more important for public health decision-making because they directly quantify the preventable proportion of risk factors. In clinical nursing practice, although patient admission assessments include items on the history of alcohol consumption and dizziness/vertigo, the potential interaction between these factors and their specific impact on fall risk has not been thoroughly examined.

This study employed RERI, API, and SI to validate whether an additive interaction exists between a history of alcohol consumption and symptoms of dizziness/vertigo, and to quantify the impact of this interaction on fall risk among individuals with mental disorders.

## 2 Methods

### 2.1 Study design

This study is a cross-sectional study, with data collected from wards of a mental health center between June 14, 2023, and December 15, 2024. The study was conducted in accordance with the Strengthening the Reporting of Observational Studies in Epidemiology (STROBE) guidelines for reporting cross-sectional studies.

### 2.2 Participants

The participants in this study were patients who had been admitted to the hospital with mental disorders. The inclusion criteria were as follows (1): has been given a diagnosis of mental disorders by the International Statistical Classification of Diseases and Health-Related Problems, Tenth Revision (ICD-10), encompassing conditions such as depression, anxiety disorders, and schizophrenia (2); age≥18 years, or <18 years if accompanied by a parent; (3) being able to provide a comprehensive clinical history; (4) having a family member who can respond on behalf of the patient in cases of language impairment; and (5) voluntary signing of informed consent by both patients and their families. The exclusion criteria were: (1) patients in the acute phase of their mental disorder; or (2) the presence of acute illness or medical history that significantly increases the risk of falls, such as acute cardiovascular events or stroke, or the withdrawal stage of substance use disorder; or (3) moderate-to-severe cognitive dysfunction.

### 2.3 Data source

In this study, data on multiple variables were extracted from the hospital’s electronic medical record system to ensure accuracy and completeness. The primary outcome measure was the incidence of falls. The World Health Organization defines a fall as an event in which a person inadvertently comes to rest on the ground, floor, or another lower level. Falls, trips, and slips can occur on one level or from a height (22). In this study, we specifically focused on falls on level ground (i.e., falls occurring at the same level, including trips and slips) and excluded data on falls from heights (e.g., falls from stairs, beds, or other elevated surfaces). We collected patients’ falls that occurred at least once in the past 6 months based on nursing records or self-reports from patients and their families at admission. The remaining variables were collected in two parts. One part was assessed based on the patient’s current condition based on the patient’s current condition at admission, including body mass index(BMI), comorbidities (e.g., diabetes mellitus, hypertension), and diagnoses. The other part was determined by researchers based on standardized assessments administered at admission, which explicitly inquired about the patient’s condition prior to the fall. This data included family accompaniment, the patient’s bedridden status, history of alcohol consumption and smoking, and presence of dizziness/vertigo, among other factors.

The collected data included:

Demographics: including gender, age, accompaniment by a family member, and diagnostic type. Patients were categorised into six age groups, based on WHO and Chinese standards: minors (<18 years), young adults (18–44 years), middle-aged adults (45–59 years), young elderly (60–74 years), mid elderly (75–84 years), and late elderly (≥85 years).

Anthropometrics: BMI was calculated as weight (kg)/height (m)^2^, and adults were categorised as underweight with a BMI <18.5 Kg/m^2^, normal weight with a BMI <24 Kg/m^2^, overweight with a BMI <28 Kg/m^2^, and obese with a BMI ≥28 Kg/m^2^ (23). Minor participants were classified into four categories: underweight, normal weight, overweight, and obese according to age and standard deviation using the WHO ‘5–19 years growth reference data’ (24).

Comorbidities and Clinical Status: the presence of comorbidities (e.g., diabetes mellitus, hypertension), the patient’s bedridden status, and whether the patient had undergone Modified Electroconvulsive Therapy (MECT) within the preceding week were documented. Bedridden status was operationally defined as a physician’s order for complete bed rest or documentation in the nursing record that the patient required assistance for all transfers (e.g., from bed to chair) due to a significant coexisting physical condition, rather than a primary psychiatric symptom.

Substance Use History: considering clinical relevance and reliability in retrospective data collection, a history of alcohol consumption was defined as drinking ≥4 times per week for at least 1 year, and a history of smoking was defined as smoking ≥1 cigarette per day for at least 6 months (25).

Medication Usage: including benzodiazepines, antiepileptic drugs, antihypertensive drugs, antipsychotics, hypoglycemic agents, anesthetics, and mood stabilizers. To ensure a consistent and mutually exclusive classification, medications were categorized based on their primary pharmacological class rather than their clinical indication in individual patients. The “anesthetics” category specifically refers to the use of intravenous anesthetic agents (e.g., propofol) for procedural sedation during MECT, not for general surgical purposes.

#### 2.3.1 Assessment tools

Dizziness/Vertigo: given that vertigo represents a specific subtype of dizziness (26), clinical evaluations typically do not distinguish between these two conditions. Therefore, the Chinese version of the Vertigo Syndrome Scale (VSS-C) was used to assess it. This scale consists of 34 items and has demonstrated good reliability and validity (27). VSS-C comprises two subscales: the Vertigo Symptoms Scale (VSS-VER) and the Autonomic Anxiety Symptoms Scale (VSS-AA). Each item is scored on a scale from 0 to 4, with a total score ranging from 0 to136. And higher total scores indicate a more severe degree of vertigo experienced by the participant. The VSS-VER subscale includes 19 items, specifically items 1a to 1e, 4, 5, 7a to 7e, 11, 15, and 18a to 18e. In this study, only the VSS-VER subscale was evaluated, as it was utilized exclusively to ascertain the presence of dizziness/vertigo symptoms among participants. A score exceeding 0 points was indicative of dizziness/vertigo.

Anxiety: was assessed using the Generalized Anxiety Disorder-7 (GAD-7) scale, where each item is scored from 0 to 3 points, resulting in a total score range of 0 to 21 points. Higher scores reflect more severe anxiety symptoms. Specifically, scores ranging from 0 to 4 points indicate the absence of anxiety, scores from 5 to 9 points suggest mild anxiety, scores from 10 to 15 points denote moderate anxiety, and scores from 16 to 21 points indicate severe anxiety (28). In the current study, scores greater than 4 points were classified as indicative of the presence of anxiety.

Depression: was conducted using the Patient Health Questionnaire–2 (PHQ-2) (29). This scale consists of two items, each evaluated on a scale from 0 to 3. A score of 0 denotes the complete absence of symptoms, 1 points indicates the presence of symptoms on a few days, 2 points indicates the presence of symptoms on more than half of the days, and 3 points reflects the presence of symptoms nearly every day. The total score ranges from 0 to 6, with a score of 3 or higher suggesting depressive symptoms.

### 2.4 Sample size calculation

The sample size estimation was informed by Vander’s (30) study. Assuming a statistical power of 0.80 and a bilateral alpha level of 0.05, the sample size was determined based on the probability of the outcome in the doubly unexposed reference group (p00), the marginal prevalence of the first exposure (P(G = 1)), the marginal prevalence of the second exposure (P(E = 1)), the odds ratio describing the dependence between the two exposures, and the probability of the outcome in the doubly unexposed reference group (p00). The minimum required sample size was calculated to be 2063, and 2210 were finally included in the study.

### 2.5 Quality control

Data collection in the study was carried out by 27 nursing staff who had received unified professional training. Upon completion of the survey, the quality of the results was promptly evaluated by specialized personnel, ensuring that any missing information was supplemented and finalized. Data entry was performed independently by two nurses to guarantee accuracy and completeness. To prevent the duplication of research participants, particularly in the case of repeated hospitalizations, the research team implemented a systematic approach for tracking and documenting participants. Specifically, we developed a central, shared digital screening log that was accessible to all 27 data collectors. The staff member must check the patient’s unique Medical Record Number against this central log prior to any data collection. The log contained a comprehensive list of all patients who had been previously screened or enrolled. If a patient’s Medical Record Number was found in the log, they were not considered for re-enrollment, ensuring that each individual was represented only once in the final study sample.

### 2.6 Statistical analysis

Statistical analyses were conducted using SPSS version 26.0. Variables with missing data of less than 1% were filled in using the mean value. Categorical data were presented as frequencies and percentages (n, %), and metrological data of non-normal distribution are shown as the Median and interquartile range (*M*, *IQR*). Group comparisons were made using the chi-square (*χ*²) test. The Chi-square test for trend (The trend *χ²* test)was used to examine trends in qualitative data. Binary logistic regression analysis was utilized to evaluate the independent associations of factors such as dizziness/vertigo and the history of alcohol consumption with the incidence of falls. The variance inflation factor was calculated to assess potential multicollinearity issues in the variables, and the *Hosmer*-*Lemeshow* test was used to check the fit of the model. To account for potential interactions between variables, interaction terms (e.g., dizziness/vertigo × alcohol consumption) were incorporated to assess the multiplicative interaction effect. There is a multiplicative interaction when the product term coefficient of the logistic regression model ≠ 0 and is statistically significant. The estimates of covariate parameters and the covariance matrix from the logistic regression model were obtained. To assess additive interaction indices, namely the relative excess risk due to interaction (RERI), the attributable proportion due to interaction (API), and the synergy index (SI), calculations were performed using the Excel spreadsheet developed by Andersson (31) et al. The employed formulas were: RERI = *OR*_11_ - *OR*_10_ - *OR*_01_ + 1; API = RERI/*OR*_11_; SI = (*OR*_11_ - 1)/(*OR*_01_ - 1), where *OR*_11_ represents the odds ratio when both risk factors are present, and *OR*_10_ and *OR*_01_ represent the odds ratios when each risk factor is present individually. And then, 95% confidence intervals (*CI*s) for the RERI and API were computed. An additive interaction effect between the two factors was indicated when the 95% CIs for the RERI and API did not encompass 0, and the 95% *CI*s for the SI did not include 1 (31). A synergistic interaction was inferred when both RERI and API exceeded 0, and SI was greater than 1, whereas an antagonistic interaction was suggested by the opposite conditions. All statistical analyses were conducted with a significance level of *α* = *0.05* (*P* < 0*.05*). The effect levels of all variables were estimated by the 95% confidence interval (95% *CI*).

### 2.7 Ethics statement

The study was approved by the hospital’s Ethics Committee and was conducted following relevant ethical standards(2024-LUNSHENXINZIDI-068). All participants were recruited voluntarily and provided written informed consent before participation. During the analysis, all personal information of the patients was anonymized.

## 3 Results

### 3.1 Characteristics of included patients

A total of 2,210 subjects were included in the study, with an observed incidence of falls at 8.78%. The Median (*IQR*) age was 41 (38) years. The diagnoses among participants included cognitive impairment(162, 7.33%), schizophrenia spectrum disorders (767, 34.71%), mood disorders (629, 28.46%), other organic mental disorders (166, 7.51%), neurotic, stress-related and somatoform disorders(172, 7.78%), and other mental disorders, such as eating disorders (314, 14.21%)(**Supplementary Table S1**). Of these, 1,034 (46.79%) were male, while 1,176 (53.21%) were female. The Median (*IQR*) age of individuals in the falls group was 30 (51) years, and 124 (63.9%) were female. The three most prevalent diagnoses, ranked in order, were mood disorders at 70(36.08%), schizophrenia spectrum disorders at 36(18.56%), and other organic mental disorders at 32(16.49%). A statistically significant difference was observed between the two groups (*P* <.05) concerning gender, family accompaniment, body mass index (BMI), a history of alcohol consumption, use of antiepileptic drugs, symptoms of dizziness/vertigo, diagnostic type, and use of mood stabilizers (**Table 1** and **Supplementary Table S2**).

**Table 1:** Characteristics of the participants [n,(%)](n=2210).

Variables	Total group (n=2210)	Fall group(n=194)	Non-fall group(n=2016)	*χ^2^*	*P*-value
Gender				9.789	0.002^**^
male	1034(46.79)	70(36.08)	964(47.82)		
female	1176(53.21)	62(63.92)	1052(52.18)		
family accompaniment				14.88	<.001^***^
yes	1206(54.57)	142(73.20)	1064(52.78)		
no	1004(45.43)	52(26.80)	952(47.22)		
alcohol consumption				11.307	<.001^***^
no	1992(90.14)	156(80.41)	1836(91.07)		
yes	218(9.86)	38(19.59)	180(8.93)		
depression				3.714	0.054
no	1372(62.08)	108(55.67)	1264(62.70)		
yes	838(37.92)	86(44.33)	752(37.30)		
mood stabilizers				5.468	0.019^*^
no	1678(75.93)	134(69.07)	1544(76.59)		
yes	532(24.07)	60(30.93)	472(23.41)		
antiepileptic drugs				15.211	<.001^***^
no	2046(92.58)	166(85.57)	1880(93.25)		
yes	164(7.42)	28(14.43)	136(6.75)		
disease duration				6.814	0.078
≤5year	1170(52.94)	116(59.80)	1054(52.28)		
6-10year	440(19.91)	38(19.59)	402(19.94)		
11-15year	146(6.61)	6(3.10)	140(6.94)		
>15year	454(20.54)	34(17.51)	420(20.84)		
diagnostic type				55.447	<.001^***^
cognitive impairment	162(7.33)	10(5.16)	152(7.54)		
schizophrenia spectrum disorders	767(34.71)	36(18.56)	731(36.26)		
mood disorders	629(28.46)	70(36.08)	559(27.73)		
neurotic, stress-related and somatoform disorders	172(7.78)	26(13.40)	146(7.24)		
other organic mental disorders	166(7.51)	32(16.49)	134(6.65)		
other mental disorders	314(14.21)	20(10.31)	294(14.58)		
BMI group				13.289^a^	<.001^***^
underweight	256(11.58)	46(23.71)	210(10.41)		
normal weight	1122(50.77)	108(55.70)	1014(50.30)		
overweight	562(25.43)	28(14.40)	534(26.49)		
obese	270(12.22)	12(6.19)	258(12.80)		
dizziness/vertigo				49.754^a^	<.001^***^
no	1410(63.80)	60(30.93)	1350(66.97)		
yes	800(36.20)	134(69.07)	666(33.03)		

a : The trend *χ*^2^ test, χ^2^: Chi-square value

^*^P<0.05; ^**^P<0.01; ^***^P<0.001

### 3.2 Results of multifactorial regression of risk factors for falls in patients with mental disorders

A binary multiple logistic regression model was developed, with the patient’s history of falls within the past six months as the dependent variable, and age, gender (coded as female=0, male=1), family accompaniment, use of antiepileptic drugs, history of alcohol consumption, presence of dizziness/vertigo, use of mood stabilizers, diagnostic type (coded as schizophrenia spectrum disorders=0, cognitive impairment=1, mood disorders=2, neurotic, stress-related and somatoform disorders=3, other organic mental disorders=4 and other mental disorders=5) and BMI group (coded as normal weight=0, underweight=1, overweight=2, and obese=3) as the independent variables. In the multiple regression analysis, after controlling for the variables of age, gender, family accompaniment, and mood stabilizer use, several factors were identified as independent risk factors for falls in patients with mental disorders. These factors included the use of antiepileptic drugs (*OR* = 4.011, 95% *CI*: 2.296-7.007, *P* <.001), a history of alcohol consumption (*OR* = 2.251, 95% *CI*: 1.474-3.437, *P* <.001), dizziness/vertigo (*OR* = 4.163, 95% *CI*: 2.867-6.045, *P* <.001) and body mass index (BMI) classifications including underweight (*OR* = 1.681, 95% *CI*: 1.128-2.503, *P* = 0.011), overweight (*OR* = 0.579, 95% *CI*: 0.366-0.916, *P* = 0.020), and obesity (*OR* = 0.496, 95% *CI*: 0.257-0.958, *P* = 0.037). Additionally, among patient diagnostic types, neurotic, stress-related, and somatoform disorders(OR = 1.856, 95% CI: 1.025-3.361, *P* = 0.041), as well as other organic mental disorders(OR = 3.087, 95% CI: 1.649-5.780, *P* <.001), were associated with fall risk (**Table 2**). There was no collinearity between the independent variables. And the Hosmer-Lemeshow test indicated a good fit model(*P* = 0.193).

**Table 2:** Binary multiple logistic regression analysis of risk factors for falls in patients with mental disorders(n=2210).

Variables	*B*	SE	*Wald χ*2	*P-value*	*OR^#^*(95%*CI*)
antiepileptic drugs	1.389	0.285	23.817	<.001^***^	4.011 (2.296-7.007)
alcohol consumption	0.811	0.216	14.125	<.001^***^	2.251(1.474-3.437)
dizziness/vertigo	1.426	0.190	56.170	<.001^***^	4.163(2.867-6.045)
BMI	*Ref*	*Ref*	19.086	<.001^***^	*Ref*
BMI (1)	0.519	0.203	6.525	0.011^*^	1.681(1.128-2.503)
BMI(2)	-0.547	0.234	5.455	0.020^*^	0.579(0.366-0.916)
BMI(3)	-0.701	0.336	4.360	0.037^*^	0.496(0.257-0.958)
Diagnostic type	*Ref*	*Ref*	39.509	<.001^***^	*Ref*
Diagnosis(1)	-0.346	0.430	0.648	0.421	0.707(0.304-1.644)
Diagnosis(2)	-0.213	0.267	0.639	0.424	0.808(0.479-1.363)
Diagnosis(3)	0.618	0.303	4.168	0.041^*^	1.856(1.025-3.361)
Diagnosis(4)	1.127	0.320	12.413	<.001^***^	3.087(1.649-5.780)
Diagnosis(5)	-0.59	0.328	3.229	0.072	0.554(0.291-1.055)

B: regression coefficients, SE: standard error, OR: Odds Ratio, CI: Confidence Interval

^#^: adjusted by age, gender, family accompaniment, and use of mood stabilizers

*Ref*: reference group.

BMI(1): underweight

BMI(2): overweight

BMI(3): obese

Diagnosis(1): cognitive impairment

Diagnosis(2): mood disorders

Diagnosis(3): neurotic, stress-related and somatoform disorders

Diagnosis(4): other organic mental disorders

Diagnosis(5): other mental disorders

^*^P<0.05; ^**^P<0.01; ^***^P<0.001

### 3.3 Analysis of the multiplicative interaction of dizziness/vertigo and a history of alcohol consumption with the occurrence of falls in patients with mental disorders

The history of falls within 6 months was used as the dependent variable, and dizziness/vertigo, a history of alcohol consumption, and dizziness/vertigo×history of alcohol consumption were included as independent variables in the logistic regression model. The findings of the study indicated the presence of a multiplicative interaction between dizziness/vertigo and a history of alcohol consumption on the occurrence of falls, after controlling for confounding variables such as age, gender, family accompaniment, BMI, presence of antiepileptic drugs, and use of mood stabilizers (*P* <.05) (**Table 3**).

**Table 3:** Analysis of the multiplicative interaction between dizziness/vertigo and history of alcohol consumption on the occurrence of falls in patients with mental disorders(n=2210).

Variables	*B*	SE	*Wald χ* ^2^	*P-value*	*OR*^#^(95%*CI*)
alcohol consumption	0.053	0.449	0.014	0.706	1.055(0.437-2.544)
dizziness/vertigo	1.268	0.201	39.634	<.001^***^	3.553(2.395-5.273)
alcohol consumption×dizziness/vertigo	1.084	0.516	4.414	0.036^*^	2.957(1.076-8.129)

B: regression coefficients, SE: standard error, OR: Odds Ratio, CI: Confidence Interval

^#^: adjusted by age, gender, family accompaniment, BMI, use of antiepileptic drugs, and use of mood stabilizers

^*^P<0.05; ^***^P<0.001

### 3.4 Crossover analysis of dizziness/vertigo and a history of alcohol consumption with incident falls

Following adjustment for confounders, it was observed that patients with mental disorders who experienced both dizziness/vertigo and had a history of alcohol consumption exhibited an 11.082-fold increased risk of falls compared to those without dizziness/vertigo and a history of alcohol consumption (95% *CI*: 6.368-19.285, *P* <.001). Dizziness/vertigo and a history of alcohol consumption were transformed into dummy variables in the regression model, and the three indicators assessing additive interaction, along with their 95% *CI*, were calculated using the formula. The results revealed that the RERI, API, and SI were 7.740 (95% *CI*: 2.701-12.239), 0.674(95% *CI*: 0.511-0.838), and 3.864(95% *CI*: 2.040-7.320), respectively, after adjusting for the relevant variables (**Table 4**).

**Table 4:** Additive interaction analysis between dizziness/vertigo and history of alcohol consumption on the occurrence of falls in patients with mental disorders(n=2210).

Dizziness/Vertigo	Alcohol Consumption	Fall/Total	Univariate analysis			Multivariate analysis		
			*OR*	95%*CI*	*P*-value	*OR* ^#^	95%*CI*	*P*-value
no	no	54/1282	1.00			1.00		
yes	no	102/710	3.815	2.705-5.381	<.001^***^	3.553^***^	2.395-5.273	<.001^***^
no	yes	6/128	1.118	0.472-2.653	0.800	1.055	0.437-2.544	0.746
yes	yes	32/90	12.547	7.531-20.903	<.001^***^	11.082^***^	6.368-19.285	<.001^***^
		*RERI*	7.470	2.701-12.239				
		*API*	0.674	0.511-0.838				
		*SI*	3.864	2.040-7.320				

OR: Odds Ratio, CI: Confidence Interval

^#^: adjusted by age, gender, family accompaniment, BMI, use of antiepileptic drugs, and use of mood stabilizers;

RERI: the relative excess risk due to interaction; API: the attributable proportion due to interaction; *SI*: the synergy index

RERI = *OR*_11_ - *OR*_10_ - *OR*_01_ + 1; *API* = *RERI*/*OR*_11_; *SI* = (*OR*_11_ - 1)/(*OR*_01_ - 1)

^***^P<0.001

## 4 Discussion

This study offers novel exploratory insights into the etiology of falls among individuals with psychiatric disorders, especially on the potential interaction between a history of alcohol comsumption and dizziness/vertigo. Our data confirm the prevalence of falls in this population (9) (8.78%, 11.68% in the elderly subgroup) and the influence of traditional factors like antiepileptic drugs. It further suggests that individual factors’ cumulative effect may not fully explain fall - risk composition. This is a preliminary attempt to quantify the interaction between these two characteristics in fall occurrence. Our analysis shows that the excess risk of their coexistence may exceed the sum of individual effects. This finding provides a promising direction for future research to identify potential high - risk subgroups and needs further validation.

First, our multivariate analysis identified several independent predictors. Consistent with established pharmacologic properties, antiepileptic drugs were associated with increased fall risk, attributed to sedation and ataxia resulting from suppressed central nervous system excitability (32). Regarding diagnostic categories, patients with neurotic, stress-related, and somatoform disorders, and other organic mental disorders exhibited significantly higher risks than those with schizophrenia spectrum disorders. This may be because patients with neurotic, stress-related, and somatoform disorders frequently use benzodiazepines, which impair psychomotor function and balance (33), while those with other organic mental disorders often involve structural brain damage impairing gait regulation and judgment (34). Conversely, the lower risk observed in the schizophrenia spectrum disorders group may stem from increased tolerance to antipsychotic medications or reduced physical activity due to negative symptoms. Interestingly, using normal BMI as a reference, we observed that underweight status increased fall risk, whereas overweight and obesity acted as protective factors. This phenomenon may be attributed to age- and sex-specific associations between weight gain and falls (35), which were not analyzed in this study. Notably, 55.67% of the patients who experienced falls were taking benzodiazepines, yet this factor was not retained as an independent predictor in the multivariable model. After ruling out potential collinearity, we hypothesize a possible mediating effect. Dizziness/vertigo is a strong independent predictor of falls. Maihoub et al (36). used objective posturography to demonstrate that dizziness/vertigo reflects measurable deficits in balance control. Since dizzinesss/vertigo is also a common side effect of benzodiazepines, the direct effect of this drug was significantly weakened when more direct predictors were incorporated into multivariate models.

Interaction analysis points toward a positive multiplicative and additive interaction between a history of alcohol consumption and dizziness/vertigo. When both factors coexist, the risk of falls is 3.864 times (95% *CI*: 2.040-7.320) greater than the combined effects of the two factors when present independently. Within the co-exposed group, 67.4% (95% *CI*: 0.511–0.838) of falls could be attributed to the interaction between a history of alcohol consumption and dizziness/vertigo. The fall incidence resulting from the synergistic interaction of these two factors may be 7.470 times greater (95% CI: 2.701–12.239) than that attributable to other factors. Although the small sample size in the co-exposure group (n=32) warrants cautious interpretation, the observed effect trend aligns with neurophysiological hypotheses. From a biological perspective, this synergy may stem from concurrent impairment of compensatory reserves within the cerebellar-vestibular network. It is known that chronic alcohol consumption induces cerebellar atrophy and impairs motor coordination through oxidative damage and the toxicity of metabolites such as acetic acid (37). Moreover, this long-term damage to the brain structure may persist even after abstinence (38). Dizziness/vertigo in patients with mental disorders typically stems from vestibular dysfunction or altered functional connectivity in motor processing regions such as the anterior insula (39). When both factors coexist, the alcohol-damaged cerebellum may lose its compensatory capacity for acute vestibular signals (dizziness/vertigo), synergistically exacerbating balance impairment and leading to postural control failure. Additionally, alcohol consumption induces vasoconstriction and atherosclerosis (40), thereby increasing the risk of orthostatic hypotension and exacerbating the severity of dizziness/vertigo episodes, creating an unstable vicious cycle. This synergistic effect explains why the combined risk exceeds the simple sum of individual exposures. Only 32 patients in this study had both a history of alcohol consumption and dizziness/vertigo symptoms, limiting the feasibility of stratified analysis. However, analysis of diagnostic composition revealed that 75% of patients in the co-exposure group were diagnosed with depressive episodes. We propose that this phenomenon may stem from specific pathophysiological processes. First, individuals experiencing depressive episodes are concurrently exposed to two key risk factors: anhedonia drives patients to use alcohol as a coping mechanism, and somatization symptoms may directly manifest as dizziness/vertigo. Second, chronic alcohol consumption exacerbates the course of depression (41), thereby reinforcing substance use behavior. Patients ultimately exhibit a high fall risk due to multiple impairments in cognition, motor function, and sensation. This finding suggests that the combination of alcohol history and dizziness/vertigo may constitute a high-risk fall phenotype among patients with mental disorders, with those exhibiting depressive symptoms warranting particular clinical attention.

Against this backdrop, if our findings are validated in large cohort studies, they may offer new perspectives for fall prevention. First, current psychiatric fall risk scales (e.g., Wilson-Sims Fall Risk Assessment Tool, WSFRAT; Edmundson Psychiatric Fall Risk Assessment, EPFRA) typically aggregate risk factors cumulatively (42, 43). These tools may underestimate the true risk for co-exposed patients, as they potentially fail to account for the interactions identified in our study. Second, theoretically, mitigating a single factor can reduce the synergistic component of risk, thereby offering a high-yield fall prevention strategy. For instance, while a patient’s alcohol history may be difficult to alter, symptoms of dizziness/vertigo represent a potentially modifiable target. That is, for patients with co-exposed risk factors, healthcare providers prioritizing interventions such as medication adjustments, installing glare-free lighting systems, or enhancing psychological interventions, which may reduce the risk of falls among individuals with mental disorders. Finally, even though further validation is required, these findings align with plausible neurophysiological mechanisms. Healthcare providers could maintain clinical vigilance toward patients exhibiting this ‘high-risk-for-falling profile’.

This study has limitations. First, the accuracy of self-reporting of fall history and dizziness/vertigo symptoms is susceptible to memory bias or interference from hallucinations and delusions, leading to biased data collection. Secondly, the study was constrained by a limited sample size, with only 32 cases documented in the co-exposure group (alcohol consumption combined with dizziness/vertigo). This resulted in excessively wide confidence intervals and precluded further stratified analysis. This small subsample size makes our interaction findings inherently exploratory and limits their generalizability. Additionally, the study did not collect data on the duration of alcohol consumption, the amount of alcohol intake, functional status, or the frequency of dizziness/vertigo episodes among patients. As a result, it was not feasible to elucidate the dose-response relationship between these two factors and fall risk, nor to determine the critical threshold at which the risk of falls significantly increases.

## 5 Conclusions

Our research indicates that a history of alcohol consumption and dizziness/vertigo symptoms were independent predictors of the occurrence of falls in patients with mental disorders. More importantly, our analysis suggests that when these two factors coexist, they may increase fall risk through potential synergistic effects, with their combined impact exceeding the sum of their individual risks. Although exploratory in nature, these findings offer novel insights into the relationship between substance use history, physical symptoms, and patient safety, laying the groundwork for future investigations into this topic. Given the limitations of this study, future research should validate these interactions in larger prospective cohorts to explore the scientific basis for fall prevention strategies targeting this specific subgroup.

## Data Availability

The original contributions presented in the study are included in the article/supplementary material. Further inquiries can be directed to the corresponding author.
