# Supplementary material for: Interaction effects of alcohol consumption and dizziness/vertigo on fall risk in psychiatric Inpatients: a cross-sectional study
Source: Front Psychiatry. 2026 Jan 8;16:1653281. doi: 10.3389/fpsyt.2025.1653281 (PMC12823966; doi:10.3389/fpsyt.2025.1653281)
Supplement: Supplementary file 1 [file Table1.docx]

## Supplementary Tables

Supplementary Table S1 Classification Scheme of Mental Disorders Included in the Study(n=2210)

| **Diagnostic Category(n)** | **Included Specific Diagnoses**  **（ICD-10 Code Range）** | **n(%)** |
| --- | --- | --- |
| **Cognitive Impairment**  **(162)** | Alzheimer’s disease dementia(F00) | 150(6.78) |
|  | Vascular-type dementia(F01) | 12(0.53) |
| **Other Organic Mental Disorders(166)** | Post-traumatic psychosis of the brain(F06.802) | 38(1.72) |
|  | Mental disorders due to physical condition(F06.808) | 39(1.75) |
|  | Mental disorders due to cerebrovascular disease(F06.809) | 89(4.03) |
| **Schizophrenia spectrum disorders(767)** | Schizophrenia(F20) | 646(29.23) |
|  | Acute transient psychotic disorde(F23) | 88(3.97) |
|  | Hallucinatory Delusional State(F28.x02) | 33(1.48) |
| **Mood Disorders(629)** | Bipolar disorder(F31) | 76(3.44) |
|  | Manic episode(F30) | 32(1.44) |
|  | Depressive episode(F32) | 451(20.41) |
|  | Recurrent depressive disorder(F33) | 70(3.17) |
| **Neurotic, stress-related and somatoform disorders(172)** | Anxiety disorder(F41.900) | 109(4.92) |
|  | Dissociative Disorders(F44) | 25(1.13) |
|  | Somatoform disorders(F45.900) | 38(1.71) |
| **Other mental disorders(314)** | Mental and behavioral disorders due to psychoactive substance use (F10-F19) | 82(3.71) |
|  | Eating disorders(F50.900) | 8(0.35) |
|  | Hyperkinetic disorders(F90) | 62(2.81) |
|  | Habit and Impulse Disorders(F63) | 12(0.54) |
|  | behavioral disorders with onset usually occurring in childhood and adolescence(F98.900) | 126(5.70) |
|  | Childhood Mood Disorder(F93.900) | 24(1.09) |

Supplementary Table S2 Characteristics of the participants［n,( %)］(n=2210)（*P*＞0.1）

| variables | total group  （n=2210） | Fall group（n=194） | Non-fall group（n=2016） | *χ^2^* | *P*-value |
| --- | --- | --- | --- | --- | --- |
| **smoking** |  |  |  | 0.448 | 0.503 |
| no | 1794(81.18) | 154(79.38) | 1640(81.35) |  |  |
| yes | 416(18.82) | 40(20.62) | 376(18.65) |  |  |
| **anxiety** |  |  |  | 0.091 | 0.590 |
| no | 1442(65.25) | 130(67.01) | 1312(65.08) |  |  |
| yes | 768(34.75) | 64(32.99) | 704(34.92) |  |  |
| **MECT** |  |  |  | 1.840 | 0.175 |
| no | 1936(87.60) | 164(84.54) | 1772(87.90) |  |  |
| yes | 274(12.40) | 30(15.46) | 244(12.10) |  |  |
| **bedridden status** |  |  |  | 0.907 | 0.341 |
| no | 2124(96.11) | 184(94.85) | 1940(96.23) |  |  |
| yes | 86(3.89) | 10(5.15) | 76(3.77) |  |  |
| **hypertension** |  |  |  | 1.584 | 0.208 |
| no | 1784(80.72) | 150(77.32) | 1634(81.05) |  |  |
| yes | 426(19.28) | 44(22.68) | 382(18.95) |  |  |
| **diabetes mellitus** |  |  |  | 0.172 | 0.679 |
| no | 1986(89.86) | 176(90.72) | 1810(89.78) |  |  |
| yes | 224(10.14) | 18(9.28) | 206(10.22) |  |  |
| **benzodiazepines** |  |  |  | 2.691 | 0.101 |
| no | 1104(49.95) | 86(44.33) | 1018(50.50) |  |  |
| yes | 1106(50.05) | 108(55.67) | 998(49.50) |  |  |
| **antihypertensive drugs** |  |  |  | 1.584 | 0.208 |
| no | 1784(80.72) | 150(77.32) | 1634(81.05) |  |  |
| yes | 426(19.28) | 44(22.68) | 382(18.95) |  |  |
| **anesthetics** |  |  |  | 0.060 | 0.807 |
| no | 2104(95.20) | 184(94.85) | 1920(95.24) |  |  |
| yes | 106(4.80) | 10(5.15) | 96(4.76) |  |  |
| **antipsychotics** |  |  |  | 0.898 | 0.343 |
| no | 422(19.10) | 42(21.65) | 380(18.85) |  |  |
| yes | 1788(89.90) | 152(78.35) | 1636(81.15) |  |  |
| **hypoglycemic agents** |  |  |  | 1.172 | 0.679 |
| no | 1986(89.86) | 176(90.72) | 1810(89.78) |  |  |
| yes | 224(10.14) | 18(9.28) | 206(10.22) |  |  |
| **age group** |  |  |  | 0.438a | 0.508 |
| minors | 390(17.65) | 66(34.02) | 324(16.07) |  |  |
| young adults | 792(35.84) | 44(22.68) | 748(37.10) |  |  |
| middle-aged | 446(20.18) | 16(8.25) | 430(21.33) |  |  |
| young elderly | 322(14.57) | 38(19.59) | 284(14.09) |  |  |
| mid elderly | 170(7.69) | 20(10.31) | 150(7.44) |  |  |
| late elderly | 90(4.07) | 10(5.15) | 80(3.97) |  |  |
| **education** |  |  |  | 1.734a | 0.188 |
| primary school degree | 438(19.82) | 34(17.52) | 404(20.04) |  |  |
| junior middle school degree | 710(32.13) | 72(37.11) | 638(31.64) |  |  |
| high-school degree | 516(23.34) | 58(29.90) | 458(22.72) |  |  |
| junior college education degree or above | 546(24.71) | 30(15.47) | 516(25.60) |  |  |

^a^: The trend *χ*² test
